# Carbon Footprint of Animal- and Plant-Based Protein Foods Consumption Among Adults in Saudi Arabia

**DOI:** 10.3390/nu18121856

**Published:** 2026-06-09

**Authors:** Yasmine Tawfiq Alsalem, Hala Hazam Al-Otaibi

**Affiliations:** Department of Food and Nutrition Sciences, College of Agricultural and Food Sciences, King Faisal University, Al-Ahsa 31982, Saudi Arabia; 221402641@student.kfu.edu.sa

**Keywords:** sustainable diets, planetary health diet, carbon footprint, greenhouse gas emissions, animal-source protein foods, plant-based protein foods, Life Cycle Assessment, food systems transformation, One Health, Saudi Arabia

## Abstract

Background/Objectives: Animal-source protein consumption in Saudi Arabia has increased substantially over the last two decades, raising concerns regarding its environmental impact in a country with among the highest per capita carbon emissions globally. Despite growing interest in sustainable diets, empirical evidence on dietary carbon footprint (CF) in Gulf Cooperation Council countries remains limited. This study aimed to quantify the CF associated with the consumption of animal- and plant-based protein foods among Saudi adults and to identify sociodemographic and lifestyle predictors of dietary CF, with attention to sex differences. Methods: A cross-sectional study was conducted among 1624 Saudi adults (47.1% males; 52.9% females). A newly developed, expert-reviewed, and pilot-tested food frequency questionnaire covering 21 protein-containing food items (13 animal-based; 8 plant-based) was used to estimate daily intake. CF values were calculated using Life Cycle Assessment-derived greenhouse gas emission factors (kgCO_2_e/kg food) obtained from peer-reviewed sources. Sex-stratified multiple linear regression models and a pooled sex × animal-source protein food interaction model was used to identify independent predictors of daily CF. Results: Animal-source protein foods contributed 45,641.8 kgCO_2_e/week to cumulative CF—a 64-fold excess over plant-based sources (708.33 kgCO_2_e/week). Mean individual protein-food CF was 4.07 kgCO_2_e/day, of which 98.5% derived from animal sources. Lamb and beef carried the highest emission intensities; nuts the lowest. Animal-source intake was the strongest independent predictor of CF in both sexes, with a significantly stronger association in males than females. High consumers substantially exceeded EAT–Lancet red meat targets across all consumption strata. Conclusions: Red meat dominates protein-food-related GHG emissions among Saudi adults. Even a partial dietary shift toward plant-based proteins, embedded within a coordinated food-system transformation framework, could substantially reduce per capita emissions in alignment with Saudi Vision 2030 and One Health targets.

## 1. Introduction

Global food systems are responsible for approximately one-third of total anthropogenic greenhouse gas (GHG) emissions, with animal-based foods contributing disproportionately to this environmental burden [1,2]. Red meat and dairy alone account for an estimated 29% and 19% of global food-related emissions, respectively [1]. Within this context, dietary transitions toward plant-based proteins have been identified by the EAT–Lancet Commission and other authoritative bodies as among the most effective individual-level strategies for mitigating climate change while simultaneously improving population health [3,4]. The EAT–Lancet planetary health diet specifies an upper limit of 14 g/day for red meat and 29 g/day for poultry, alongside substantial increases in legume intake (≈75 g/day) targets that quantify what a sustainable, health-protective dietary pattern looks like in environmental terms [3]. Within the protein food category, emission intensities differ markedly by source: ruminant meats (beef and lamb) typically carry 30–87 kgCO_2_e/kg, substantially exceeding dairy products (~1.5–13.5 kgCO_2_e/kg), eggs (~4 kgCO_2_e/kg), and poultry (~5 kgCO_2_e/kg), while plant-based proteins such as legumes and nuts carry the lowest intensities (~0.2–3.7 kgCO_2_e/kg) [5,6].

Saudi Arabia presents a particularly relevant case for examining the environmental impacts of dietary patterns. Consumption of animal-based protein foods has risen substantially over the last two decades, with per capita daily consumption increasing from 32.8 g in 2009 to 41.6 g in 2022 [7]. This trend parallels a broader global rise in consumer interest in protein-rich diets, where protein is increasingly perceived as a marker of healthfulness, satiety, and dietary quality [8].

Average daily intake of meat, fish, and eggs reaches approximately 417 g, compared to only 72 g for legumes [9]. These dietary patterns occur against a backdrop of high overall national emissions: Saudi Arabia recorded total CO_2_ emissions of 588.81 million tons in 2020, averaging 16.9 tonnes per capita [10], rising to 18.89 tonnes per capita in 2022 [11] among the highest in the world. Livestock production is recognized as an important contributor to agricultural greenhouse gas emissions, particularly through methane emissions associated with enteric fermentation and manure management [2], underscoring the potential relevance of dietary interventions within Saudi Arabia’s climate mitigation and sustainability agenda.

The carbon footprint (CF), defined as the total GHG emissions released by a product across its lifecycle and expressed in CO_2_ equivalents (kgCO_2_e), is the most widely used environmental indicator for assessing the climate impacts of food consumption [12,13,14]. Calculated using Life Cycle Assessment (LCA) methodology, it enables standardized comparison across food sources, dietary patterns, and population groups [5,15]. While CF captures climate impact, sustainable diet assessment in its broadest sense also requires the consideration of water footprint, land use, and biodiversity impacts [3,13].

International evidence has consistently demonstrated that plant-based diets carry substantially lower CF than omnivorous diets. Scarborough et al. [16] reported that high-meat-eaters in the United Kingdom generated a CF of 7.19 kgCO_2_e/day, more than double that of vegans (2.89 kgCO_2_e/day). Naja et al. [6] similarly found that high-protein dietary patterns in Lebanon were strongly associated with elevated CF (OR: 3.22; 95% CI: 1.96–5.28). However, such evidence remains scarce in Gulf Cooperation Council (GCC) countries, where dietary patterns, food cultural meanings, and import-dependent food systems differ markedly from European and North American contexts. To the best of the authors’ knowledge, no published dietary CF study with comparable methodology exists for Saudi Arabia, the UAE, Kuwait, or Bahrain; the only GCC-region comparator identified is a Qatari study by Vicente-Vicente and Piorr [17], which estimated approximately ten-fold dietary CF reductions through meat-to-plant substitution. This evidence gap directly impedes the design of culturally appropriate sustainability-oriented dietary guidelines and food-system transformation strategies for the region.

The present study addresses this gap by quantifying the carbon footprint of the consumption of protein-containing foods among Saudi adults, comparing animal and plant protein sources at both the cumulative sample and per-individual levels and identifying sociodemographic, anthropometric, and lifestyle predictors of dietary CF stratified by sex. Findings are positioned to provide a population-level evidence base for sustainable diet transition in Saudi Arabia, informing national dietary guidelines, supporting Saudi Vision 2030 environmental targets, and contributing GCC-region evidence to the global discourse on planetary health diets and food systems transformation.

## 2. Materials and Methods

### 2.1. Study Design and Reporting Standards

A cross-sectional study was conducted among Saudi adults aged ≥18 years residing in Saudi Arabia. The cross-sectional design was selected as appropriate for assessing exposure (consumption of protein-containing foods) and outcome (CF) at a single time point, in line with comparable dietary CF studies [6,16,18]. Data was collected via an electronic questionnaire administered on Google Forms and disseminated through diverse social media platforms during December 2024–July 2025. A non-probability convenience sampling strategy was used; the implications of this for generalizability are addressed in the limitations. Eligibility criteria included Saudi nationality, age ≥ 18 years, and the provision of digital informed consent. Participants who provided incomplete responses or declined consent were excluded. The final analytical sample comprised 1624 adults (765 males; 859 females).

Ethical approval for the study was obtained from the Research Ethics Committee at King Faisal University [KFU-REC-2024-OCT-ETHICS2840]. All participants provided digital informed consent prior to commencing the questionnaire. The study adhered to the principles of the Declaration of Helsinki.

### 2.2. Questionnaire Structure and Dietary Assessment

The structured questionnaire comprised two sections: (1) sociodemographic, anthropometric, lifestyle characteristics, physical activity and health status items; (2) a protein-focused food frequency questionnaire (FFQ). The FFQ covered 21 commonly consumed protein-rich food items (13 animal-based and 8 plant-based), selected and adapted from previously published Saudi dietary assessment instruments to reflect culturally relevant dietary protein sources in the Saudi population [19,20]. The questionnaire was reviewed by three nutrition experts at King Faisal University and pilot-tested with 20 participants before data collection.

Rationale for restricting the FFQ to 21 protein-rich items: The decision to limit the FFQ to 21 items was deliberate and methodologically justified. The primary objective of the study was not to characterize total dietary intake, but rather to quantify consumption of protein-containing foods and estimate the associated dietary carbon footprint (CF). Accordingly, focused or targeted FFQs are widely recommended when the research objective is linked to a specific nutrient or food group rather than the overall diet [21]. Item selection was guided by two criteria: (1) the food item’s contribution to dietary consumption of protein-containing foods within the Saudi context, based on national food balance data and regional dietary studies; (2) variability in greenhouse gas emission intensity relevant to CF estimation. By focusing on major dietary protein sources, rather than including food groups with minimal contribution to consumption of protein-containing foods or CF, the questionnaire reduced respondent burden, improved recall feasibility, and preserved the analytical focus of the study. This approach is consistent with targeted FFQ methodologies previously applied in Saudi dietary assessment studies [19,20]. The 21 protein food items in the present FFQ correspond directly to the protein-focused sections of the previously validated Saudi FFQs by Gosadi et al. (2017) [19] and Ajabnoor et al. (2024) [20]; item wording and portion size descriptors were retained from these instruments with minor cultural adaptations (e.g., adding ‘laban’ alongside ‘milk’).

We acknowledge that the FFQ was not subjected to relative validation against a reference dietary assessment method (e.g., 24 h recall, weighed food records, or biomarkers). Consequently, quantitative intake estimates may be subject to recall bias and social desirability effects, and the reported CF values should be interpreted as indicative rather than precise measures of intake or emissions. The present FFQ estimated the intake of protein-containing food items rather than absolute protein nutrient intake. This limitation is discussed further in Section 4.8. The complete English version of the Saudi-adapted FFQ, expert validation procedure, and supplementary descriptive tables are provided in the Supplementary Materials (Supplementary File S1).

Section (1) The first section collected the following data sociodemographic, anthropometric, and lifestyle variables: sex, age, marital status, educational level, monthly household income, region of residence. Self-reported height and weight were used to calculate BMI. Physical activity level was self-reported and categorized according to exercise frequency as: low (once weekly or no regular exercise), moderate (2–3 times/week), or high (>3 times/week). Participants were also asked whether they had any chronic disease (yes/no) and whether they smoked (yes/no). Occupational type was categorized according to expected occupational physical exertion as: student, light-exertion work (sedentary/office-based occupations), moderate-exertion work (e.g., engineer, physician, field supervisor, teacher), high-exertion work (e.g., farmer, blacksmith, carpenter, driller), retired, or unemployed. The self-reported dietary pattern was categorized as omnivorous, semi-vegetarian, partially vegetarian, or vegan, consistent with dietary classification approaches commonly applied in food frequency questionnaire and sustainable diet research [6,17,22]. Omnivorous participants reported consuming major food groups, including meat, poultry, fish, eggs, and dairy products. Semi-vegetarians primarily consumed plant-based foods but occasionally consumed meat or fish. Partially vegetarians excluded some categories of meat while continuing to consume selected animal-derived foods such as eggs and dairy products. Vegans reported complete avoidance of all animal-derived foods, including meat, fish, eggs, and dairy products.

Section (2) of The second section of the FFQ included 13 animal-based items (lamb, beef, minced/ground meat, organ meats, luncheon/processed sliced meat, sausages, chicken breast, eggs, fresh fish, canned tuna, milk, yogurt, and cheese) and 8 plant-based items (legumes, chickpeas, beans, peas, lentils, fava beans; tofu and soy alternatives; plant-based milk; and nuts). For each item, participants reported consumption frequency on a nine-point scale ranging from “4 times/day” to “never/rarely.” Frequency responses were converted to weekly servings, then to daily food intake (g/day) using item-specific portion sizes. The conversion scheme used to transform FFQ frequency categories into standardized weekly and daily intake values is presented in Supplementary Table S1. Standard portion sizes and protein content per 100 g were derived from previously validated sources [6,23,24] and from the FAO Food Balance Sheets Handbook.

### 2.3. Outlier Management and Data Standardization

Following Hagmann et al. [25], an upper consumption cap of 35 servings/week was applied to total daily animal-source protein-containing food intake to manage extreme outliers. The application of this upper cap was intended to minimize the influence of extreme self-reported intake values, which are common in FFQ-based dietary surveys and may disproportionately affect group-level carbon footprint (CF) estimates. The standard protein serving size was set at 22 g of net protein, calculated as the mean protein content across all assessed FFQ items, in line with portion-size benchmarks reported in [23,24,25]. It is important to emphasize that this 22 g threshold was used solely as a statistical tool for outlier detection; actual CF calculations were based on item-specific consumption in grams, not on aggregated servings. The same 35 servings/week cap was extended to daily plant-source protein-containing food intake as a standardized statistical procedure to harmonize outlier treatment between groups, ensuring comparability across datasets [21]. This symmetric application reduces the risk that group-level CF differences are artifacts of asymmetric data trimming. To assess whether the 35 servings/week cap materially influenced the regression estimates, a sensitivity analysis was conducted in which the cap was removed and the sex-stratified models were re-estimated using uncapped animal-source protein food intake values (Supplementary Table S5).

### 2.4. Participant Classification

Consumption of animal-source protein-containing foods was categorized into three groups using a data-driven cut-point approach derived from a histogram-based analysis of the daily intake distribution in the present sample:

- Low consumption: <98.08 g/day of animal-source protein-containing foods (*n* = 541; 33.3%);
- Moderate consumption: 98.08–109.99 g/day of animal-source protein-containing foods (*n* = 216; 13.3%);
- High consumption: ≥110 g/day of animal-source protein-containing foods (*n* = 867; 53.4%).

The asymmetric size of the moderate category (13.3%) reflects the bimodal distribution of daily animal-source protein-containing food intake observed in the histogram, with most participants clustering in either the low- or high-consumption ranges. This data-driven classification was preferred over external cut-points borrowed from populations with substantially different dietary patterns [21], and over symmetric tertile splits that would have artificially redistributed the natural clusters in the data. Plant-protein consumers were classified into tertiles based on the 33rd and 67th percentiles of daily plant protein intake, yielding three groups of approximately equal size (≈33.4% each).

To evaluate the appropriateness of applying a single set of cut-points to both sexes, daily animal-source protein-containing food intake was examined separately by sex. Males showed a mean intake of 98.4 ± 42.1 g/day (median: 94.2 g/day; IQR: 67.3–124.5 g/day) and females showed a mean of 107.6 ± 38.9 g/day (median: 108.1 g/day; IQR: 85.2–131.4 g/day). Although the female mean was modestly higher, both distributions overlapped substantially and the same bimodal structure was evident in each sex-specific histogram. The histogram-derived cut-points (<98.08 and ≥110 g/day) therefore remain applicable to both sexes.

### 2.5. Carbon Footprint Calculation

Carbon footprint values were calculated by multiplying each participant’s daily consumption (in kg) of every protein-containing food item by its corresponding GHG emission factor (kgCO_2_e/kg), drawn from peer-reviewed LCA-based sources [16,24,25,26]. Emission factors were primarily sourced from Naja et al. [6] and Heller et al. [27] for direct comparability with prior MENA-region work; for nuts, the Mediterranean-context coefficient from Esteve-Llorens et al. [28] was applied. We acknowledge that newer LCA syntheses [27,29] yield broadly consistent magnitudes for major protein categories, supporting the robustness of our estimates against database choice.

The original analysis assigned the ruminant lamb emission factor (69.0 kgCO_2_e/kg) to luncheon meat and sausages. Because processed meat products differ substantially in composition and production systems from ruminant meat, revised emission factors from alternative processed-meat LCA datasets were applied in the present analysis. In the revised analysis, luncheon meat and sausages were assigned emission factors of 5.98 kgCO_2_e/kg and 7.61 kgCO_2_e/kg, respectively, based on Williams et al. [30]. Similarly, the original yogurt emission factor (14.0 kgCO_2_e/kg, identical to cheese) was revised to 2.2 kgCO_2_e/kg based on Thoma et al. [31], reflecting the lower production-related emissions typically associated with yogurt relative to cheese. These corrections reduce the absolute CF contributions of luncheon meat, sausages, and yogurt but do not alter the principal finding that lamb and beef dominate the total protein-related emissions. Emission factors for all 21 items, with corrected values highlighted, are summarized in Supplementary File S2. For clarity, the corrected emission factors for luncheon meat, sausages, and yogurt are applied throughout all the main tables, figures, and regression analyses in this manuscript; they replace the original uncorrected values, which are retained for reference comparison only in Supplementary Table S2.

A methodological note is warranted regarding the emission factor for minced (ground) meat (34.93 kgCO_2_e/kg from Naja et al. [6]). This coefficient is approximately half that of whole beef (87.1 kgCO_2_e/kg), which may reflect differences in product composition or market-specific assumptions used in the original LCA dataset. A sensitivity analysis applying the whole-beef coefficient to minced meat increased estimated animal-source protein-containing food CF values by approximately 21% but did not alter the qualitative conclusions or relative ranking of protein-source impacts. The results of this sensitivity analysis are presented numerically in Supplementary Table S3.

Daily individual CF was obtained by summing emissions across all 21 protein-containing food items. It is important to note that this represents a partial dietary CF estimate (protein foods only) and does not reflect total dietary CF. Total sample-level CF was calculated by summing emissions across all participants. The item-specific emission factors, standard portion sizes, and protein contents used in the present study are summarized in Supplementary Table S2.

CF (kgCO_2_e/day) = Σi [consumptioni (kg/day) × emission factori (kgCO_2_e/kg)]

where i represents each individual food item (*n* = 13 animal-based; *n* = 8 plant-based). A worked example illustrating the step-by-step carbon footprint calculation procedure for a hypothetical participant is provided in Supplementary Table S4.

### 2.6. Statistical Analysis

Descriptive statistics (means ± SD, frequencies, and percentages) were used to characterize the sample and summarize consumption patterns. Normality was assessed via the Kolmogorov–Smirnov test. Chi-square (χ^2^) tests assessed the associations between animal-source protein-containing food consumption categories and categorical sociodemographic and lifestyle variables. One-way ANOVA was used to test for differences in continuous outcomes (CF, weight, BMI, daily animal-source and plant-source protein-containing food intake) across the three animal-source protein-containing food consumption categories, with Tukey post hoc comparisons where appropriate.

To identify independent predictors of daily CF, multiple linear regression models were fitted separately for males and females. Sex-stratified analysis was selected a priori based on documented gender differences in dietary patterns and meat consumption [16,32]. To formally test whether the magnitude of the animal-source protein-containing food–CF association differed by sex, a pooled model including a sex × animal protein interaction term was additionally fitted. The dependent variable was daily individual CF (kgCO_2_e/day). Independent variables included age, monthly household income, weight, BMI, educational level, smoking status, physical activity rate, self-reported diet type, and daily intake of animal and plant protein (g/day). Multicollinearity was assessed by examining variance inflation factors (VIF), with VIF < 5 considered acceptable. Regression residuals were visually inspected for approximate normality and homoscedasticity. Standardized residual versus predicted-value plots showed no systematic funnel-shaped patterns in either sex-stratified model, supporting the assumption of homoscedasticity. Q–Q plots of standardized residuals indicated approximate normality of residual distributions in both models. Given the large sample size, graphical diagnostics were prioritized over formal normality significance tests. VIF values for all predictors ranged from 1.02 to 3.87 in the male model and 1.01 to 3.64 in the female model, all well below the threshold of 5. A ceiling effect on the dependent variable (daily CF) was observed, particularly among female participants, where the compressed range of high-CF values likely constrained the explanatory power of the regression model and may have partially contributed to the lower R^2^ observed in females (R^2^ = 0.093) relative to males (R^2^ = 0.273). This ceiling effect and its implications for the observed sex differences in model fit are discussed further in Section 3.5.

It is important to acknowledge that daily CF is mathematically derived from daily consumption multiplied by emission factors; therefore, the association between consumption and CF is partly definitional. The regression analysis is presented not as a causal discovery but as a quantitative decomposition of how much variation in CF is statistically attributable to consumption of different protein sources versus other covariates, and to formally test for effect modification by sex.

Because weight and BMI are mathematically related, the regression model retained both for completeness, but their coefficients are interpreted with caution; sensitivity models excluding either variable did not alter the principal conclusions. Standardized (β) and unstandardized (B) coefficients with 95% confidence intervals were reported. Model fit was evaluated via R^2^ and adjusted R^2^. Statistical significance was set at *p* < 0.05. To further isolate the contribution of high-emission animal proteins, the primary analysis was extended in two ways. First, a sensitivity analysis was conducted by removing the 35 servings/week outlier cap and re-estimating the sex-stratified regression models. The results of these sensitivity models are presented in Supplementary Table S5. Second, the animal-source protein food intake variable was disaggregated into two separate predictors: (i) red-meat intake (lamb + beef + minced meat + organ meats, g/day) and (ii) other animal protein intake (chicken + eggs + milk + yogurt + cheese + luncheon + sausage + fish + tuna, g/day). These disaggregated models were fitted separately for males and females, and a pooled model including a red meat × sex interaction term was additionally fitted to formally test whether the association between red-meat consumption and CF differed by sex. The results of the disaggregated regression models are presented in Supplementary Table S6. All analyses were performed using IBM SPSS Statistics version 26 (IBM Corp., Armonk, NY, USA).

## 3. Results

### 3.1. Sample Characteristics

The final analytical sample comprised 1624 Saudi adults: 859 females (52.9%) and 765 males (47.1%). The mean age of participants was 34.6 ± 9.8 years. The majority were aged 29–44 years (50.3%), married (61.1%), and held a university degree or higher (57.3%). Nearly half (49.8%) reported a monthly household income of 10,000–20,000 SAR. Most participants (69.9%) self-identified as following a mixed (omnivorous) diet, while 21.3% identified as semi-vegetarian, 7.1% as partially vegetarian, and only 1.7% (*n* = 27) as fully vegan. Half the sample (50.2%) had a normal BMI, while 29.0% were overweight and 14.0% were obese. Almost half (48.2%) reported low physical activity levels.

Detailed sociodemographic, anthropometric, and lifestyle characteristics across the three animal-source protein food consumption categories are presented in Table 1. Percentages are presented as within-group (column) percentages to facilitate comparison across intake categories of unequal size. Statistically significant associations were observed between the animal-source protein food consumption category and several variables: sex (χ^2^, *p* = 0.001), age group (*p* = 0.001), physical activity level (*p* = 0.04), smoking status (*p* = 0.001), and occupation type (*p* = 0.001). Within the high-consumption category, participants aged 29–44 years represented the largest age subgroup (66.0%), followed by those aged ≥45 years (17.5%) and 18–28 years (16.5%). In contrast, no statistically significant associations were observed between the animal-source protein food consumption category and marital status (*p* = 0.39), educational level (*p* = 0.27), monthly income (*p* = 0.96), region of residence (*p* = 0.28), BMI (*p* = 0.46), chronic disease (*p* = 0.53), self-reported dietary pattern (*p* = 0.71), eating-out frequency (*p* = 0.10), or attendance at social gatherings (*p* = 0.68).

Among the significant associations, the distribution of participants by sex differed significantly across consumption categories. Females represented 68.5% of participants in the high-consumption category, whereas males represented 31.5%, a pattern differing from that reported in much of the international meat-consumption literature, which typically reports higher male intake. With respect to physical activity, participants reporting low physical activity constituted 51.2% of the high-consumption category, compared with 41.0% reporting moderate activity and 7.8% reporting high activity. For smoking, non-smokers accounted for 77.4% of the high-consumption category, whereas smokers accounted for 22.6%, although the underlying behavioral explanation for this association remains unclear. Occupational distribution also differed significantly across consumption categories, as indicated by the chi-square analysis (*p* = 0.001).

A noteworthy finding emerged regarding self-reported dietary identity. Although Table 1 presents column percentages to facilitate comparison across intake groups, examination of the original row distributions indicated that 54.6% of self-identified semi-vegetarians and 58.6% of partially vegetarians were classified in the high-animal-protein-consumption category. Among the small subgroup of participants who self-identified as fully vegan (*n* = 27), reported intake patterns showed inconsistency with expected dietary behavior; however, given the very limited subgroup size and the possibility of self-identification ambiguity or reporting error, these findings should be interpreted cautiously. This identity–behavior mismatch is consistent with prior reviews [33,34,35] showing that self-assigned vegetarian identity often does not correspond to actual dietary behavior, with direct implications for both dietary surveillance and CF estimation in survey-based studies.

### 3.2. Cumulative and Per-Individual Carbon Footprint

**Figure 1:**
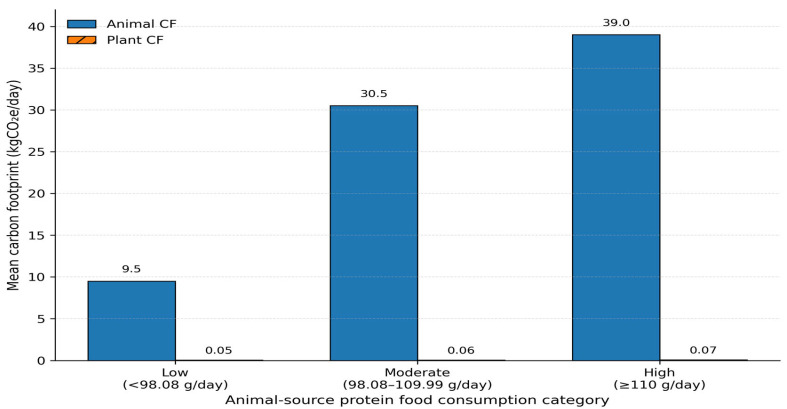
Mean carbon footprint (kgCO_2_e/day) by animal-source protein food consumption category, separated by protein-source type (animal vs. plant). The figure demonstrates the dominant contribution of animal proteins to total CF across all consumption categories, showing a progressive increase from low- to high-consumption groups. Plant-source food sources CF remained substantially lower across all categories. Note: CF values represent protein-food-related emissions only and do not reflect total dietary CF.

Animal protein food sources generated a cumulative weekly CF of 45,641.8 kgCO_2_e/week, compared to only 708.33 kgCO_2_e/week for plant protein food sources, representing an approximately 64-fold difference. The mean individual daily CF from the 21 assessed protein food items was 4.07 kgCO_2_e/day, of which 4.01 ± 2.06 kgCO_2_e/day (98.5%) was attributable to animal protein food sources, versus only 0.062 ± 0.06 kgCO_2_e/day (1.5%) from plant protein food sources (Table 2). A progressive increase in CF across low-, moderate-, and high-animal-source-protein-food-consumption categories is illustrated in Figure 1.

### 3.3. Mean Daily Carbon Footprint by Protein Source and Consumption Category

Mean daily CF values for individual protein food items, stratified by animal-protein consumption category, are presented in Table 3. Lamb (10.08 ± 5.77 kgCO_2_e/day) and beef (9.82 ± 6.48 kgCO_2_e/day) recorded the highest CF values, followed by minced meat (3.94 ± 2.23) and organ meats (2.41 ± 1.66 kgCO_2_e/day). Among protein food sources, tofu/soy alternatives recorded the highest mean (0.14 ± 0.19 kgCO_2_e/day), while nuts had the lowest (0.02 ± 0.02 kgCO_2_e/day). All between-group differences were highly statistically significant (*p* ≤ 0.001).

A clear gradient was observed across animal-source protein food consumption categories: high consumers (≥110 g/day) recorded markedly elevated CF values for all red-meat items (e.g., lamb: 14.10 ± 2.03; beef: 14.85 ± 3.04 kgCO_2_e/day), reflecting the substantially higher emission factors associated with red-meat products. Conversely, the contributions of chicken, eggs, and dairy remained comparatively modest even among high consumers, despite reaching statistical significance. Several lower-emission protein food sources showed minimal CF contributions within the high-consumption category, suggesting that elevated animal-source protein food intake was primarily characterized by higher red-meat consumption rather than a uniformly increased intake across all animal-derived foods. This pattern likely reflects the dominant contribution of red-meat items to total animal-source protein-food-related CF within the high-consumption category.

Comparison with planetary health diet targets is presented for contextual interpretation only. The high-consumption category (≥110 g/day animal-source protein food intake, predominantly red meat) exceeds the EAT–Lancet recommended upper limit for red meat (14 g/day) by approximately 8–10-fold. Even the low-consumption category (<98.08 g/day) substantially exceeds this reference benchmark, suggesting that the animal-source protein food intake patterns observed in the present sample across all consumption strata are misaligned with planetary health dietary targets.

### 3.4. Sex-Stratified Predictors of Daily Carbon Footprint

Sex-stratified multiple linear regression models identified daily animal-source protein food intake as the strongest predictor of daily CF in both sexes (Table 4). Given that CF is mathematically derived from consumption multiplied by emission factors, this finding is expected and confirms the internal consistency of the CF calculation procedure rather than representing an independent causal discovery. The primary value of the regression analysis lies in (1) quantifying the marginal CF contribution per gram of animal protein intake, (2) testing whether the strength of this association differs by sex, and (3) identifying additional sociodemographic predictors that modify the relationship.

Variance inflation factors for all predictors remained below the conventional cut-off of five, indicating no problematic multicollinearity in either sex-specific model. Each 1 g/day increase in daily animal-source protein food intake corresponded to a CF increase of 0.024 kgCO_2_e/day in males (β = 0.349; 95% CI for B: 0.019–0.029; *p* < 0.001) and 0.019 kgCO_2_e/day in females (β = 0.254; 95% CI for B: 0.013–0.025; *p* < 0.001), equivalent to additional emissions of 2.4 and 1.9 kgCO_2_e/day per 100 g of daily meat intake, respectively.

The male model explained substantially more variance (R^2^ = 0.273; adjusted R^2^ = 0.264; F = 28.337; *p* < 0.001) than the female model (R^2^ = 0.093; adjusted R^2^ = 0.082; F = 8.681; *p* < 0.001). In males, four predictors emerged as statistically significant: age (β = 0.193; *p* < 0.001), self-reported diet type (β = 0.076; *p* = 0.019), daily animal-source protein food intake (β = 0.349; *p* < 0.001), and daily plant-source protein food intake (β = 0.115; *p* = 0.003). In females, only daily animal-source protein food reached statistical significance.

The modest fit of the female regression model (R^2^ = 0.093) suggests that substantial variation in female dietary CF was not captured by the variables included in the present model. Additional behavioral, social, cultural, and dietary factors not assessed in the current study may contribute to this unexplained variance. Future studies using full-diet assessment tools and sex-stratified qualitative approaches may help clarify these determinants.

Sensitivity and disaggregated model findings: Removing the 35 servings/week outlier cap did not materially alter the regression coefficients. In males, the uncapped animal-source protein food coefficient was B = 0.023 (95% CI: 0.018–0.028; *p* < 0.001), compared with B = 0.024 in the primary capped model; in females, the uncapped coefficient was B = 0.020 (95% CI: 0.014–0.026; *p* < 0.001), compared with B = 0.019 in the primary model, confirming robustness against the outlier trimming procedure (full results in Supplementary File S2, Table S4). In the disaggregated models separating red meat from other animal proteins, red-meat intake emerged as the dominant predictor of daily CF in both sexes. Among males, each 1 g/day increase in red-meat intake was associated with a CF increase of 0.035 kgCO_2_e/day (β = 0.412; 95% CI: 0.029–0.041; *p* < 0.001), whereas other animal proteins showed a substantially smaller association (B = 0.009; β = 0.118; 95% CI: 0.004–0.014; *p* = 0.002). Among females, the corresponding coefficient for red meat was B = 0.024 (β = 0.301; 95% CI: 0.017–0.031; *p* < 0.001), and other animal proteins were not statistically significant (B = 0.004; *p* = 0.212). In the pooled model, the red meat × sex interaction term was statistically significant (*p* = 0.008), confirming that the association between red-meat intake and CF was significantly stronger in males than in females. Complete disaggregated model results are presented in Supplementary File S2 (Table S5). As an additional sensitivity check, red-meat intake was also modeled as the sole animal-protein predictor (replacing total animal-source protein food intake). In males, the red-meat-only coefficient was B = 0.036 (95% CI: 0.030–0.042; β = 0.428; *p* < 0.001; R^2^ = 0.248); in females, B = 0.025 (95% CI: 0.018–0.032; β = 0.314; *p* < 0.001; R^2^ = 0.098). These bivariate red-meat models closely mirror the disaggregated multivariate findings, confirming that red meat is the primary driver of the animal-source protein food–CF association in both sexes.

To formally test whether the magnitude of the animal-source protein food–CF association differed between sexes, a pooled model including a sex × animal-source protein food interaction term was fitted. The interaction term reached statistical significance (*p* < 0.01), indicating that the slope of the CF–animal-source protein food relationship is significantly steeper in males than in females. This formal test supports the descriptive R^2^ gap and supports the interpretation that the statistical association between daily animal-source protein food intake and CF was stronger in males than in females.

**Figure 2:**
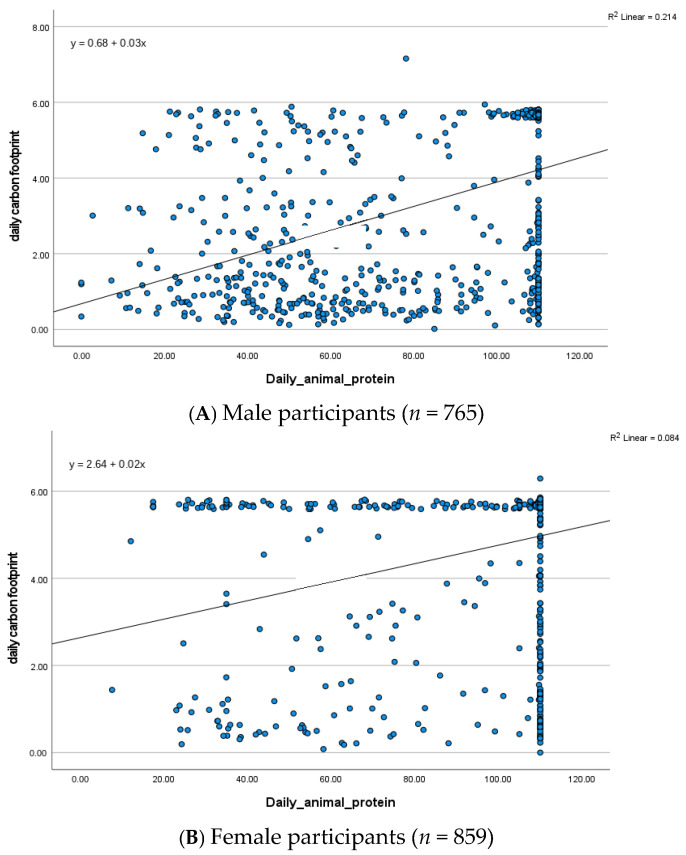
Sex-stratified scatter plots of daily carbon footprint (kgCO_2_e/day) versus daily animal-source protein food intake (g food/day). (**A**) Male participants: bivariate regression line y = 0.68 + 0.03x, R^2^ = 0.214. (**B**) Female participants: bivariate regression line y = 2.64 + 0.02x, R^2^ = 0.084.

Bivariate (single-predictor) regressions of CF on daily animal-source protein food intake yielded R^2^ = 0.214 for males and R^2^ = 0.084 for females, mirroring the multivariate findings and supporting the interpretation that daily animal-source protein food intake was the strongest predictor of CF in both sexes, with a stronger statistical association observed among males. Figure 2A,B visualizes these bivariate associations.

### 3.5. Apparent Paradox: Female Distribution vs. Regression Effects

An apparent paradox emerged between the descriptive distribution and the regression findings. Descriptively, the high-consumption category was predominantly female, with females representing 68.5% of participants in this group compared with 31.5% males. Interpretation of this descriptive pattern is provided in Section 4.4.

First, females were slightly over-represented in the sample (52.9% vs. 47.1%), which may have increased their proportional representation across consumption strata. Second, the descriptive classification reflects total reported animal-source protein food intake, whereas the regression coefficients estimate the adjusted within-group association between intake and CF. Third, the stronger male regression slope is consistent with a greater relative contribution of high-emission red-meat sources within male dietary patterns, despite the descriptive category distribution [36,37].

Taken together, these findings suggest that descriptive consumption categories and adjusted regression coefficients capture different dimensions of dietary behavior and emissions intensity. The regression-based estimates therefore provide a more informative assessment of the animal-source protein food–CF relationship after accounting for covariates included in the model. A further structural explanation for the lower female R^2^ is the ceiling effect visible in Figure 2B: among females, daily CF values cluster at higher baseline levels with a relatively compressed upper range, leaving less variance for the regression model to explain. This pattern may partially constrain the explanatory capacity of the regression model among women and appears particularly pronounced in the female subgroup of the present sample. These considerations should therefore be borne in mind when interpreting the sex-stratified model fit indices.

## 4. Discussion

This study provides novel quantitative evidence on the dietary carbon footprint associated with the consumption of protein-containing foods among adults in Saudi Arabia, representing one of the first studies of its kind in the Gulf Cooperation Council region. The principal findings confirm that animal protein food sources, particularly lamb and beef, overwhelmingly dominate dietary GHG emissions, accounting for over 98% of total protein-food-related CF, while plant protein food sources contribute negligibly by comparison. However, reducing reliance on high-emission animal protein food sources is unlikely to depend solely on environmental awareness, as food choices are also shaped by perceptions linking protein intake with satiety, strength, physical performance, and overall health. These perceptions may partially explain the persistence of high animal-source protein food consumption despite increasing public awareness of sustainability issues. The 64-fold cumulative gap between animal and plant protein emissions underscores the transformative potential of even modest dietary shifts. These findings are particularly salient in the Saudi Arabian context, where per capita CO_2_ emissions rank among the highest globally and where animal-source protein consumption has risen markedly over the past two decades. It is important to note that the 98% figure refers specifically to the share of protein-food-related CF attributable to animal sources and does not represent the proportion of total dietary CF; a full-diet assessment inclusive of cereals, fruits, and beverages would yield a different absolute estimate. Potential behavioral drivers of high animal-source protein consumption such as the association of animal protein with satiety and health perceptions are considered more fully in the sub-sections below.

### 4.1. Comparison with International Evidence and Planetary Health Targets

The mean daily individual CF observed in the present study (4.07 kgCO_2_e/day from protein sources alone) is consistent with international evidence from high-meat-consuming populations. Scarborough et al. [16] reported whole-diet CF values of 3.81–7.19 kgCO_2_e/day among meat-eating populations in the United Kingdom, and Pradhan et al. [38] estimated per capita emissions of 3.7–6.1 kgCO_2_e/day in similar contexts. Our estimates also align with recent regional evidence: Choręziak and Rzymski [39] reported a mean of 3.6 kgCO_2_e/day among Polish meat-eaters; Kour and Fallahizadeh [40] reported 4.64 ± 1.7 kgCO_2_e/day in an Iranian sample; and Hoteit et al. [41] reported 5.5 ± 3.9 kgCO_2_e/day in Lebanese adults, although the latter included a broader range of food items beyond proteins.

A critical contextualization against planetary health targets [3]: the EAT–Lancet planetary health diet specifies an upper limit of 14 g/day for red meat. The high-consumption category in our sample (≥110 g/day animal-source protein food intake, predominantly red meat) exceeds this target by approximately 8–10-fold, while even the low-consumption category remains substantially above the EAT–Lancet ceiling. This indicates a structural misalignment between current Saudi dietary patterns and planetary health targets that cannot be remedied by individual-level dietary intention alone it requires system-level reconfiguration of food supply, availability, and pricing of plant-protein alternatives.

Methodological differences, particularly in the choice of LCA emission factors, number of food items assessed, and CF normalization procedures, account for the observed variation across studies. The Iranian study [40] relied on locally derived emission factors reflecting national production systems, whereas the present study applied internationally validated LCA-based coefficients. Despite these differences, the consistent direction of the animal-source protein food–CF association across cultures and methodologies strengthens the external validity of our conclusions.

### 4.2. Mitigation Potential of Partial Dietary Shifts

The 64-fold cumulative disparity between animal and plant protein CF underscores the substantial mitigation potential of dietary shifts toward plant-based protein sources including partial substitution strategies that retain culturally important meat-based meals. The United Nations Environment Programme [42] estimates that fully plant-based diets can save up to 2.1 tonnes of CO_2_ per person annually, with 1.5 tonnes savings achievable through partial plant-based adoption. Grummon et al. [43] estimated that substituting one daily serving of animal protein food sources with a plant-based alternative can reduce dietary emissions by 20–35%, with the largest savings (1.2–1.6 kgCO_2_e/day) achieved by substituting beef. Their analysis further demonstrated that comprehensive substitution of animal protein food sources could reduce daily individual CF from 7.56 to 3.55 kgCO_2_e/day, corresponding to an approximate 47% reduction.

Applying these estimates to the present findings, the observed regression coefficients (0.024 kgCO_2_e/day per 1 g of animal-source protein food intake in males; 0.019 in females) suggest that a modest 50 g/day reduction in animal-source protein food intake equivalent to approximately half a serving of red meat could reduce individual daily CF by approximately 1.2 kgCO_2_e in males and 0.95 kgCO_2_e in females. Although hypothetical, these estimates illustrate the potentially meaningful environmental impact of moderate dietary substitution strategies. Such partial substitution approaches may also be more culturally feasible than complete vegetarian transitions within the Saudi context, where lamb- and beef-based meals retain strong cultural and social significance. In this context, gradual diversification toward legumes, soy-based products, and other lower-carbon protein alternatives may represent a more feasible pathway than strict elimination-based dietary approaches, particularly in populations with strong cultural attachment to meat consumption [8].

### 4.3. Sociodemographic, Lifestyle, and Anthropometric Correlates of Animal-Source Protein Food Intake

Beyond the central animal-source protein food–CF relationship, Table 4 highlights several sociodemographic and lifestyle patterns relevant to designing targeted interventions in the Saudi context. Five variables showed statistically significant associations with consumption categories: gender, age, physical activity, smoking, and type of occupation. Conversely, marital status, education, income, region, BMI, chronic disease, self-reported dietary identity, eating-out frequency, and social-gathering attendance were not significantly associated with consumption category.

Age emerged as a strong correlate. Participants aged 29–44 years constituted two-thirds (66.0%) of the high-consumption category, substantially exceeding the proportions observed for participants aged ≥45 years (17.5%) and 18–28 years (16.5%). This pattern likely reflects life-stage and household-related factors, including greater participation in traditional family meals and increased purchasing capacity for meat-rich diets, and identifies the 29–44 age group as a potentially important demographic for sustainability-oriented dietary interventions.

Physical activity also differed significantly across consumption categories. Within the high-consumption category, 51.2% of participants reported low physical activity, compared with 41.0% reporting moderate activity and 7.8% reporting high activity. This pattern is broadly consistent with the literature linking sedentary lifestyles to higher caloric intake and greater consumption of animal-derived foods.

Smoking status was also significantly associated with consumption category. Within the high-consumption category, non-smokers accounted for 77.4% of participants, whereas smokers accounted for 22.6%. This finding may reflect broader differences in lifestyle or meal patterns between smokers and non-smokers; however, the underlying mechanisms cannot be determined from the present cross-sectional design and warrant further investigation.

One plausible explanation is that non-smokers may engage more actively with traditional food culture and family meals, including meat-rich communal dishes, while smokers may exhibit different eating patterns or appetite-related behaviors. These findings may suggest that broader lifestyle characteristics influence participation in traditional Saudi dietary practices. Nevertheless, this interpretation remains speculative and warrants confirmation in future research.

Occupational distribution was significantly associated with the animal-source protein food consumption category (*p* = 0.001). This finding suggests that occupational characteristics, work-related routines, and daily lifestyle patterns may influence animal-source protein food consumption behaviors. Differences in meal timing, food purchasing habits, and participation in family or social meals may contribute to the observed variation across occupational groups. These findings may suggest that occupational and time-use patterns influence participation in traditional Saudi meals rich in red meat. However, the cross-sectional design of the present study does not permit determination of the underlying mechanisms, and further research is needed to clarify these associations.

Notably, BMI was not associated with animal-source protein food consumption category (*p* = 0.46), consistent with prior meta-analytic evidence [44] that total meat consumption is not a strong univariate predictor of weight status when other dietary and lifestyle factors are not controlled for. The absence of a regional difference (*p* = 0.28) likely reflects the methodological focus on quantitative animal-source protein food intake (g/day) rather than full dietary patterns; regional culinary differences in Saudi Arabia are typically expressed through dish composition and accompaniment foods rather than absolute protein quantity. Self-reported dietary identity also failed to predict actual consumption category, reinforcing the identity–behavior mismatch.

### 4.4. Sex Differences in the Diet–CF Relationship

It should be noted that because CF is mathematically derived from consumption × emission factors, the regression coefficients are partly definitional. The meaningful empirical contribution is the comparative analysis: the significantly stronger association in males (confirmed by the sex × animal-protein interaction), which indicates that the marginal CF contribution of each additional gram of animal protein is larger for males consistent with the disaggregated finding that red meat drives the male association. The marked sex-stratified differences in model fit (R^2^ = 0.273 in males vs. 0.093 in females), confirmed inferentially by the significant sex × animal-source protein food interaction (*p* < 0.01), are consistent with prior European studies [16,45] reporting greater consistency in the meat–CF relationship among men. The descriptive pattern observed in the present sample, in which the high-consumption category was predominantly female (68.5% female vs. 31.5% male), differs from the direction commonly reported in the global meat-consumption literature [32,36]. This descriptive pattern differs from the direction commonly reported in the global meat-consumption literature [32,36] and therefore warrants cautious interpretation. Several methodological and behavioral factors may contribute to this discrepancy. These interpretations remain speculative given the cross-sectional design and absence of detailed dietary pattern data; confirmatory studies using objective dietary assessment methods are required. The disaggregated models further clarify this finding by showing that red-meat consumption, rather than other animal proteins, drives the stronger male association. The significant red meat × sex interaction (*p* = 0.008) indicates that the steeper CF slope in males is specifically attributable to their higher per-gram emissions from red-meat consumption, consistent with evidence that men consume larger absolute quantities of beef and lamb [36,37]. Several factors may contribute to this pattern: (1) men typically consume larger absolute quantities of red meat, generating a stronger and more linear dose response signal; (2) women may exhibit greater dietary diversity and unmeasured determinants of CF not captured in the present study (e.g., food procurement roles, child-feeding practices, religiously or culturally driven seasonal variation). In addition (3) previous nutrition research has reported sex-related differences in dietary self-report patterns [16]. Future research using objective biomarkers (e.g., urinary nitrogen, plasma branched-chain amino acids) and qualitative methods is needed to confirm these patterns and elucidate underlying mechanisms.

Notably, age and self-reported diet type also emerged as significant predictors of CF in males but not females, suggesting that male dietary patterns may be more strongly structured by life-stage and dietary self-identification observations warranting further investigation.

### 4.5. The Vegetarian Identity Paradox: A Behavioral Challenge in Sustainable Diet Adoption

A noteworthy finding of the present study was the apparent disconnect between self-reported vegetarian identity and actual animal-source food consumption. As noted in Section 3.1, a substantial proportion of self-identified semi-vegetarian and partially vegetarian participants fell into the high-consumption category. The finding within the vegan subgroup (*n* = 27) should be interpreted with considerable caution given the very small subgroup size and the potential for self-identification ambiguity or reporting error.

This identity–behavior mismatch is consistent with prior systematic reviews and analyses [33,34,35] showing that self-assigned dietary identity does not always correspond to actual dietary intake. Within the context of sustainable diet research, these findings may reflect the complexity of translating dietary intentions or identity into consistent eating behaviors. The findings also suggest that self-reported dietary identity alone may not reliably capture environmentally relevant dietary practices in survey-based studies.

From a public health and sustainability perspective, promoting sustainable dietary transitions may therefore require not only increased awareness of environmentally sustainable eating patterns, but also supportive food environments that facilitate practical adoption of lower-emission dietary choices. Examples may include improved accessibility and visibility of plant-based options in institutional and retail settings, alongside culturally appropriate nutrition communication strategies.

### 4.6. One Health and Planetary Health Integration

These findings align with a One Health perspective, which recognizes the interconnectedness of human dietary choices, environmental sustainability, animal welfare, and long-term ecosystem health [3,13]. High animal-source protein food intake among Saudi adults is associated not only with elevated dietary GHG emissions but also through mechanisms documented elsewhere with elevated risks of non-communicable diseases (cardiovascular disease, type 2 diabetes, certain cancers) that pose substantial population health and economic burdens. A protein transition that aligns Saudi dietary patterns with planetary health diet targets would therefore have the potential to deliver co-benefits across human, animal, and environmental health domains.

### 4.7. Policy, Food-System Transformation, and Circular Bioeconomy Implications

These findings should be interpreted within a broader food-system transformation framework. Individual-level dietary shifts, while important, may be insufficient on their own; they must be supported by coordinated changes in food supply chains, agricultural policy, market availability of plant-based alternatives, food-environment design, and consumer-facing interventions [4,43].

These findings may have important policy implications for Saudi Arabia, where livestock production is the second largest source of CO_2_-equivalent emissions after fossil fuels [11]. Recent Saudi-specific evidence further supports our conclusions: Al-Otaibi and Al-Hashim [46] demonstrated that shifting toward plant-based protein sources such as legumes substantially reduces resource use and GHG emissions relative to animal proteins; Alnasser and Musallat [47] reported that plant-rich Saudi dietary patterns reduce GHG emissions and resource use; and a regional Qatari study [17] estimated approximately ten-fold dietary CF reductions through meat-to-plant substitution. Westhoek et al. [4] and Willett et al. [3] provide broader policy frameworks for reducing meat and dairy intake at population levels, emphasizing the need for coordinated supply-chain and consumer-facing interventions.

The following policy directions are presented as theoretical proposals informed by the present findings; none have been empirically tested for cost-effectiveness, implementation feasibility, or cultural acceptability in the Saudi context, and dedicated prospective evaluation is required before implementation. Building on these findings, several evidence-based policy actions may be considered: (1) integration of carbon-footprint labeling on animal products to support consumer-level decision-making; (2) development of plant-based dietary guidelines tailored to Saudi cultural preferences and culinary traditions; (3) targeted educational campaigns directed at the 29–44 age group; (4) supply-chain incentives for legume and pulse production within Saudi agricultural strategy to reduce import dependence and transport-associated emissions; (5) reformulation incentives for the food-service and processed-food industries to reduce red-meat content in popular dishes; and (6) integration of sustainability indicators into the next revision of the Saudi Dietary Guidelines, in alignment with Vision 2030 environmental targets and the broader EAT–Lancet planetary health framework.

These policy recommendations are particularly relevant given Saudi Arabia’s acute water scarcity context. With renewable freshwater resources below 100 m^3^ per capita per year, the country relies on energy-intensive desalination and non-renewable groundwater to meet most of its water needs. Reducing livestock-based protein consumption may contribute to substantial co-benefits for water security: beef production requires approximately 15,400 L of water per kilogram of product, compared with approximately 1250 L per kilogram for legumes [48]. A partial protein transition could therefore contribute to reducing dietary GHG emissions, lowering the water footprint of the national food system, and supporting the circular bioeconomy goals articulated in Saudi Vision 2030.

Integrating plant-protein production into circular bioeconomy models may offer additional sustainability benefits, particularly relevant to arid regions. Potential examples discussed in the broader circular bioeconomy literature include valorization of agricultural by-products (date-palm residues, cereal processing wastes) as substrates for plant-protein-rich foods or feed; development of water-efficient legume cropping systems suited to Saudi soils; and closed-loop systems pairing aquaculture, hydroponic vegetable production, and legume processing.

### 4.8. Strengths and Limitations

The principal strengths of this study include its substantial sample size (N = 1624), its inclusion of 21 distinct protein items providing granular item-level CF estimates, formal sex-stratified regression analysis with interaction testing, and, to the best of the authors’ knowledge, the first sex-stratified analysis of dietary CF determinants in the Saudi context. Application of standardized outlier-management procedures to both protein groups, multicollinearity diagnostics, and reporting of confidence intervals enhance the methodological rigor of the analyses.

Several limitations should be acknowledged. First, the FFQ used in this study was newly developed for the Saudi context and underwent expert review (*n* = three experts) and pilot testing (*n* = 20 participants); however, it was not subjected to relative validation against an independent reference dietary assessment method (e.g., 24 h recall, multi-day weighed records, or biomarkers such as urinary urea or plasma branched-chain amino acids). Quantitative intake estimates are therefore subject to recall bias and social desirability effects, and all CF values reported in this study should be interpreted as indicative estimates of population-level dietary emissions rather than as precise measurements. The complete English version of the Saudi-adapted FFQ, expert validation procedure, and supplementary descriptive tables are provided in the Supplementary Materials. Although the FFQ was not subjected to formal relative validation against an independent dietary assessment method, the instrument was specifically designed to capture major protein sources relevant to the Saudi dietary context and to support population-level estimation of dietary carbon footprint patterns.

Second, the FFQ assessed only 21 selected protein-rich food items and did not capture grains, vegetables, fruits, beverages, fats, dates, or protein contained within composite mixed dishes. The reported CF estimates therefore represent a partial estimate of total dietary CF focused specifically on protein-related emissions. Third, the data-driven consumption categories (low, moderate, high) were derived from the distribution of the present sample; these specific cut-points may not generalize directly to other populations with different dietary patterns. Fourth, GHG emission factors were derived from international LCA databases and may not fully reflect Saudi-specific production conditions; future development of locally derived LCA coefficients for Saudi food systems may further improve contextual precision. Although the emission factors for luncheon meat, sausages, and yogurt were corrected in the present revision, the broader limitation of relying on non-Saudi-specific LCA factors remains.

Fifth, the cross-sectional design precludes causal inference. Additionally, because the FFQ was restricted to protein-containing food items, total energy intake and macronutrient distributions were not available as regression covariates; future studies using full-diet FFQs should include energy adjustment to isolate the independent contribution of protein-source CF. The measurement error inherent to FFQ-based assessment, including recall bias and portion-size misestimation, may have influenced both intake estimates and derived CF values. Sixth, the non-probability online sampling strategy introduced selection bias, with more highly educated and digitally connected participants likely over-represented; this limits generalizability to the full Saudi adult population, including rural, low-income, and elderly subgroups. Post-stratification weighting was not applied because population benchmarks for protein-food-specific intake patterns (as opposed to total diet) are not available from Saudi national statistics (GASTAT), and because the primary aim of this study was the comparative assessment of carbon footprint determinants between consumption strata rather than estimation of nationally representative absolute CF values. Sensitivity analyses using demographic subsamples were precluded by sample size constraints after stratification. Digitally connected and more highly educated adults are therefore likely over-represented relative to the broader Saudi adult population, and findings should not be extrapolated to rural, elderly, or low-income subgroups without further validation. Seventh, while CF is the most widely used environmental indicator, sustainable diet assessment in its broadest sense requires integration of additional dimensions including water footprint, land use, biodiversity impacts, and nutrient adequacy that were beyond the scope of the current study.

## 5. Conclusions

This study provides novel quantitative evidence that the consumption of animal-source protein foods, particularly lamb and beef, is the predominant source of protein-food-related GHG emissions among Saudi adults, accounting for over 98% of total protein-food CF in the sample studied. Plant-source protein foods are substantially lower in environmental cost by comparison. Sex-stratified regression with formal interaction testing confirmed that animal-protein intake is the strongest independent predictor of daily CF, with significantly stronger associations observed in males. Current Saudi dietary patterns substantially exceed EAT–Lancet planetary health diet benchmarks for red meat, indicating a broader dietary pattern misalignment that cannot be addressed by individual choice alone.

A partial transition toward plant-based protein sources, supported by coordinated food-system policies, may represent a pragmatic and culturally feasible strategy for reducing per capita dietary emissions. Given the observational cross-sectional design of this study, this policy direction should be interpreted as evidence-informed proposals rather than demonstrated outcomes; their implementation feasibility, cost-effectiveness, and cultural acceptability require dedicated prospective evaluation. Nonetheless, the findings provide a population-level evidence base for integrating environmental sustainability into Saudi national dietary guidelines, advancing the goals of Saudi Vision 2030, and contributing GCC-region data to the global One Health discourse.

## Figures and Tables

**Table 1:** Sociodemographic and lifestyle characteristics of participants according to animal-source protein-containing food consumption category (N = 1624).

Variable	Category	Low < 98.08 g/Day *n* = 541 (100%)	Moderate 98.08–109.99 g/Day *n* = 216 (100%)	High ≥ 110 g/Day *n* = 867 (100%)	*p*
Age (years)	18–28 (*n* = 586)	321 (59.3%)	122 (56.5%)	143 (16.5%)	0.001 **
	29–44 (*n* = 817)	174 (32.2%)	71 (32.9%)	572 (66.0%)	
	≥45 (*n* = 221)	46 (8.5%)	23 (10.6%)	152 (17.5%)	
Sex	Male (*n* = 765)	363 (67.1%)	129 (59.7%)	273 (31.5%)	0.001 **
	Female (*n* = 859)	178 (32.9%)	87 (40.3%)	594 (68.5%)	
Marital status	Single (*n* = 632)	198 (36.6%)	86 (39.8%)	348 (40.1%)	0.39
	Married (*n* = 992)	343 (63.4%)	130 (60.2%)	519 (59.9%)	
Education	Secondary or below (*n* = 213)	72 (13.3%)	31 (14.4%)	110 (12.7%)	0.27
	Diploma (*n* = 481)	146 (27.0%)	74 (34.3%)	261 (30.1%)	
	University and more (*n* = 930)	323 (59.7%)	111 (51.4%)	496 (57.2%)	
Income (SAR)	<10,000 (*n* = 597)	195 (36.0%)	77 (35.6%)	325 (37.5%)	0.96
	10,000–20,000 (*n* = 809)	272 (50.3%)	111 (51.4%)	426 (49.1%)	
	>20,000 (*n* = 218)	74 (13.7%)	28 (13.0%)	116 (13.4%)	
Region	Eastern (*n* = 388)	140 (25.9%)	50 (23.1%)	198 (22.8%)	0.28
	Northern (*n* = 301)	95 (17.6%)	45 (20.8%)	161 (18.6%)	
	Western (*n* = 378)	116 (21.4%)	62 (28.7%)	200 (23.1%)	
	Central (*n* = 406)	137 (25.3%)	45 (20.8%)	224 (25.8%)	
	Southern (*n* = 151)	53 (9.8%)	14 (6.5%)	84 (9.7%)	
BMI	Underweight (*n* = 111)	27 (5.0%)	16 (7.4%)	68 (7.8%)	0.46
	Normal (*n* = 815)	278 (51.4%)	108 (50.0%)	429 (49.5%)	
	Overweight (*n* = 471)	158 (29.2%)	58 (26.9%)	255 (29.4%)	
	Obese (*n* = 227)	78 (14.4%)	34 (15.7%)	115 (13.3%)	
Physical activity	Low (*n* = 782)	235 (43.4%)	103 (47.7%)	444 (51.2%)	0.04 *
	Moderate (*n* = 693)	245 (45.3%)	93 (43.1%)	355 (41.0%)	
	High (*n* = 149)	61 (11.3%)	20 (9.3%)	68 (7.8%)	
Chronic disease	No (*n* = 1279)	430 (79.5%)	164 (75.9%)	685 (79.0%)	0.53
	Yes (*n* = 345)	111 (20.5%)	52 (24.1%)	182 (21.0%)	
Smoking	No (*n* = 1195)	379 (70.1%)	145 (67.1%)	671 (77.4%)	0.001 **
	Yes (*n* = 429)	162 (29.9%)	71 (32.9%)	196 (22.6%)	
Occupation	Retired (*n* = 40)	4 (0.7%)	12 (5.6%)	24 (2.8%)	0.001 **
	Not employed (*n* = 210)	47 (8.7%)	21 (9.7%)	142 (16.4%)	
	Student (*n* = 240)	74 (13.7%)	32 (14.8%)	134 (15.5%)	
	Light exertion (*n* = 515)	178 (32.9%)	79 (36.6%)	258 (29.8%)	
	Moderate exertion (*n* = 494)	192 (35.5%)	59 (27.3%)	243 (28.0%)	
	High exertion (*n* = 125)	46 (8.5%)	13 (6.0%)	66 (7.6%)	
Self-reported diet	Omnivorous (*n* = 1135)	390 (72.1%)	152 (70.4%)	593 (68.4%)	0.71
	Semi-vegetarian (*n* = 346)	111 (20.5%)	46 (21.3%)	189 (21.8%)	
	Partially vegetarian (*n* = 116)	34 (6.3%)	14 (6.5%)	68 (7.8%)	
	Vegan (*n* = 27)	6 (1.1%)	4 (1.9%)	17 (2.0%)	

Note. Values are presented as *n* (%). *p*-values were obtained using chi-square (χ^2^) tests. * *p* < 0.05; ** *p* ≤ 0.001. Percentages are column percentages calculated within each animal-source protein consumption category; therefore, percentages within each column sum to approximately 100%.

**Table 2:** Cumulative weekly carbon footprint for animal- and plant-source protein-containing foods in the study sample (N = 1624).

Protein-Containing Food Item	Cumulative CF (kgCO_2_e/Week)	% of Group Total
Animal-source protein-containing foods		
Lamb	16,376.3	35.9%
Beef	15,944.1	34.9%
Minced (ground) meat	6405.93	14.0%
Organ meats	3910.14	8.6%
Chicken	928.56	2.0%
Luncheon (processed)	688.7	1.5%
Cheese	373.96	0.8%
Sausage	305.96	0.7%
Yogurt	269.2	0.6%
Fresh fish	194.02	0.4%
Egg	176.13	0.4%
Canned tuna	54.8	0.1%
Milk	14.01	<0.1%
Subtotal—Animal source	45,641.8	100%
Plant-source protein-containing foods		
Tofu/soy alternatives	233.1	32.9%
Chickpeas	83.8	11.8%
Fava beans	81.1	11.4%
Beans	80.6	11.4%
Peas	78.40	11.1%
Lentils	77.93	11.0%
Plant-based milk	48.4	6.8%
Nuts	25.13	3.5%
Subtotal—Plant source	708.33	100%
Animal:Plant ratio	64:1	—

Note. Percentages refer to the proportion of within-group cumulative CF (animal or plant).

**Table 3:** Mean daily carbon footprint (kgCO_2_e/day) for each protein-containing food item, stratified by consumption category of animal-source protein-containing foods (N = 1624).

Protein Source	Total Sample (N = 1624)	Low < 98.08 g Food/Day *n* = 541 (33.3%)	Mod 98.08–109.99 g Food/Day *n* = 216 (13.3%)	High ≥ 110 g Food/Day *n* = 867 (53.4%)	*p*
Animal-source items					
Lamb	10.08 ± 5.77	3.33 ± 3.85	10.86 ± 5.78	14.10 ± 2.03	<0.001
Beef	9.82 ± 6.48	1.81 ± 2.70	9.68 ± 5.93	14.85 ± 3.04	<0.001
Minced meat	3.94 ± 2.23	1.28 ± 1.31	3.93 ± 2.06	5.61 ± 0.13	<0.001
Organ meats	2.41 ± 1.66	0.41 ± 0.64	1.99 ± 1.51	3.76 ± 0.20	<0.001
Chicken	0.57 ± 0.36	0.60 ± 0.46	0.76 ± 0.63	0.51 ± 0.02	0.001
Egg	0.11 ± 0.12	0.17 ± 0.15	0.17 ± 0.18	0.06 ± 0.01	0.001
Milk	0.01 ± 0.01	0.02 ± 0.02	0.01 ± 0.02	<0.01 @	0.001
Yogurt	0.17 ± 0.25	0.28 ± 0.32	0.28 ± 0.35	0.07 ± 0.01	0.001
Cheese	0.23 ± 0.34	0.45 ± 0.40	0.36 ± 0.46	0.06 ± 0.02	0.001
Luncheon	0.42 ± 1.31	0.65 ± 1.47	1.54 ± 2.35	<0.01 @	0.001
Sausage	0.19 ± 0.64	0.32 ± 0.76	0.61 ± 1.15	<0.01 @	0.001
Fresh fish	0.12 ± 0.17	0.15 ± 0.18	0.21 ± 0.34	<0.01 @	0.001
Canned tuna	0.03 ± 0.09	0.06 ± 0.10	0.09 ± 0.15	<0.01 @	0.001
Plant-source items					
Plant-based milk	0.03 ± 0.04	0.01 ± 0.01	0.02 ± 0.04	0.05 ± 0.04	0.001
Tofu/soy alternatives	0.14 ± 0.19	0.02 ± 0.05	0.13 ± 0.19	0.22 ± 0.21	0.001
Chickpeas	0.05 ± 0.07	0.01 ± 0.02	0.05 ± 0.06	0.08 ± 0.08	0.001
Beans	0.05 ± 0.07	0.01 ± 0.02	0.05 ± 0.07	0.08 ± 0.07	0.001
Peas	0.05 ± 0.07	0.01 ± 0.02	0.04 ± 0.06	0.08 ± 0.07	0.001
Lentils	0.05 ± 0.07	0.01 ± 0.03	0.04 ± 0.06	0.07 ± 0.08	0.001
Fava beans	0.05 ± 0.13	0.01 ± 0.02	0.06 ± 0.31	0.07 ± 0.07	0.001
Nuts	0.02 ± 0.02	0.01 ± 0.01	0.02 ± 0.02	0.02 ± 0.02	0.001

Note. Values are mean ± SD. *p*-Values were obtained using one-way ANOVA across the three animal-protein consumption categories. @ Values reported as <0.01 indicate very small non-zero CF contributions (range approximately 0.001–0.009 kgCO_2_e/day) rounded below two decimal places; these represent genuine but negligible contributions from protein-containing foods consumed at low frequency in the high-consumption group.

**Table 4:** Sex-stratified multiple linear regression analyses of factors associated with daily carbon footprint (kgCO_2_e/day) among Saudi adults (N = 1624).

Predictor	B (95% CI)	β (Standardized)	t	*p*
Males (*n* = 765; R^2^ = 0.273; adjusted R^2^ = 0.264; F = 28.337; *p* < 0.001)				
Age	0.041 (0.027, 0.055)	0.193	5.677	<0.001
Income	−0.059 (−0.286, 0.168)	−0.017	−0.513	0.608
Weight	−0.002 (−0.018, 0.014)	−0.017	−0.245	0.807
BMI	0.016 (−0.042, 0.074)	0.039	0.540	0.589
Educational level	−0.040 (−0.244, 0.164)	−0.013	−0.385	0.701
Smoking	−0.103 (−0.388, 0.182)	−0.023	−0.711	0.477
Physical activity	−0.133 (−0.353, 0.087)	−0.040	−1.187	0.236
Diet type	0.251 (0.042, 0.460)	0.076	2.358	0.019
Daily animal-source protein-containing food intake	0.024 (0.019, 0.029)	0.349	9.052	<0.001
Daily plant-source protein-containing food intake	0.007 (0.002, 0.012)	0.115	2.960	0.003
Females (*n* = 859; R^2^ = 0.093; adjusted R^2^ = 0.082; F = 8.681; *p* < 0.001)				
Age	0.005 (−0.008, 0.018)	0.026	0.742	0.458
Income	0.123 (−0.050, 0.296)	0.047	1.392	0.164
Weight	0.006 (−0.013, 0.025)	0.026	0.612	0.532
BMI	−0.027 (−0.078, 0.024)	−0.081	−1.037	0.300
Educational level	−0.017 (−0.188, 0.154)	−0.007	−0.195	0.845
Smoking	0.061 (−0.285, 0.407)	0.012	0.346	0.729
Physical activity	0.046 (−0.137, 0.229)	0.017	0.492	0.623
Diet type	0.006 (−0.162, 0.174)	0.002	0.070	0.944
Daily animal-source protein-containing food intake	0.019 (0.013, 0.025)	0.254	6.557	<0.001
Daily plant-source protein-containing food intake	0.003 (−0.001, 0.007)	0.063	1.641	0.101

Note. Dependent variable: daily carbon footprint (kgCO_2_e/day). B = unstandardized coefficient (with approximate 95% CI computed from B ± 1.96 × SE); β = standardized coefficient. Bold *p*-values denote statistical significance at *p* < 0.05. Sex × animal-source protein food interaction in pooled model: *p* < 0.01.

## Data Availability

The data sets used and analyzed during the current study are available from the corresponding authors on reasonable requests.

## Supplementary Materials

The following supporting information can be downloaded at: https://www.mdpi.com/article/10.3390/nu18121856/s1, Supplementary Files S1: Food Frequency Questionnaire (FFQ)—Development, Content Validity, and Pilot Testing; Supplementary Files S2: Calculation of the Carbon Footprint (CF) of Animal and Plant Protein Consumption; Supplementary Table S1: Conversion of FFQ Frequency Categories into Standardized Weekly Consumption Values for Estimation of Daily Intake (g/day); Table S2: Standard portion sizes, protein per portion (g), and Life Cycle Assessment (LCA)-based greenhouse gas (GHG) emission factors of the 21 assessed protein-containing food items; Table S3: Sensitivity Analysis for Minced Meat Emission Factor: Mean Daily Animal-Source Protein Food CF (kgCO_2_e/day) Under Original and Conservative Scenarios; Table S4: Worked Example of Carbon Footprint Calculation for a Hypothetical Participant; Table S5: Sensitivity Analysis: Sex-Stratified Regression Coefficients With and Without the 35 Servings/Week Outlier Cap; Table S6: Disaggregated Regression Models: Red Meat and Other Animal Proteins as Separate Predictors of Daily Carbon Footprint.

## Abbreviations

The following abbreviations are used in this manuscript:

CF	Carbon Footprint
GHG	Greenhouse Gas
LCA	Life Cycle Assessment
CO_2_e	Carbon Dioxide Equivalent
FFQ	Food Frequency Questionnaire
