# Supplementary material for: Carbon Footprint of Animal- and Plant-Based Protein Foods Consumption Among Adults in Saudi Arabia
Source: Nutrients. 2026 Jun 9;18(12):1856. doi: 10.3390/nu18121856 (PMC13305175; doi:10.3390/nu18121856)
Supplement: Supplementary file 1 [file nutrients-18-01856-s001.zip › nutrients-4344466-supplementary.pdf]

Supplementary File S1: Food Frequency Questionnaire (FFQ) – Development, Content Validity, and Pilot Testing

Title: Development and Content Validation of a Protein-Focused Food Frequency Questionnaire for the Saudi Population

1. Introduction

This supplementary document provides detailed information on the development, content validity assessment, and pilot testing of the Food Frequency Questionnaire (FFQ) used to assess protein consumption in the study titled *" Carbon Footprint of Animal and Plant Protein Consumption Among Adults in Saudi Arabia: A Cross-Sectional Study."* The FFQ was designed as a targeted instrument to capture the intake of major animal- and plant-based protein sources relevant to the Saudi dietary context, rather than to assess total dietary intake [1,2]. Because the FFQ was designed as a targeted environmental exposure assessment tool rather than a nutrient-intake quantification instrument, content validity and feasibility were prioritized in the present phase of instrument development.

2. FFQ Development

2.1. Item Selection

The initial item pool was developed based on:

- A review of previously published Saudi dietary assessment instruments [3,4].
- National food balance sheet data (FAO, 2022) to identify key protein contributors in the Saudi diet.
- Regional dietary studies from the Middle East and North Africa (MENA) region [5].
- Variability in greenhouse gas emission intensity of different protein sources to support the primary study outcome (carbon footprint estimation).

The final FFQ comprised **21 protein-rich food items**, categorized as:

- **Animal-based (13 items):** Lamb, beef, minced/ground meat products, organ meats, chicken breast/thigh, whole eggs, milk/laban, yogurt, cheese, processed luncheon meat, sausages, fresh fish/seafood, canned tuna.
- **Plant-based (8 items):** Plant-based milk, soybeans/soy alternatives (tofu), chickpeas, beans, peas, lentils, fava beans, nuts.

2.2. Frequency Scale and Portion Sizes

- **Frequency Scale:** A nine-point scale was used: *4 times/day; 3 times/day; 2 times/day; once/day; 5–6 times/week; 2–4 times/week; once/week; 1–3 times/month; never/rarely.
- **Standard Portion Sizes:** Portion sizes were defined using culturally relevant visual aids (e.g., palm-sized piece, handful, cup, container) and were derived from validated sources [5,6,7].

3. Content Validity (Expert Review)

3.1. Expert Panel
The draft FFQ was reviewed by a panel of **three nutrition experts** from the Department of Food and Nutrition Sciences at King Faisal University. The panel included specialists in dietary assessment, public health nutrition, and sustainable food systems.

3.2. Validation Criteria
Each expert independently evaluated the FFQ using a 4-point Likert scale for:

1. **Relevance:** Whether each item was relevant to measure protein intake in the Saudi population.
2. **Clarity:** Whether the wording, portion size descriptions, and frequency options were clear and unambiguous.
3. **Comprehensiveness:** Whether the 21 items adequately covered the major protein sources consumed locally.
4. **Cultural Appropriateness:** Whether the food items and portion sizes reflected actual Saudi consumption practices.

3.3. Expert Feedback and Revisions

- All three experts agreed that the 21 items were relevant and comprehensive for a *protein-focused* assessment. No major additional protein sources were recommended for inclusion. No additional protein sources were recommended for inclusion.
- Minor revisions were made to portion size descriptors:
    - *"Laban"* was added alongside *"milk"* for cultural clarity.
    - *"Hand-length portion"* was clarified for chicken breast/thigh.
    - *"Two thumbs or three teaspoons"* was added for cheese portion visualization.
- The frequency scale was confirmed as appropriate for capturing both daily and weekly intake patterns.

A Content Validity Index (CVI) was calculated at the item level (I-CVI) and scale level (S-CVI). Item-level CVI (I-CVI) was calculated as the proportion of experts rating an item as either 3 or 4 on the 4-point relevance scale, consistent with Polit and Beck [8]. Scale-level CVI (S-CVI/Ave) was computed as the average I-CVI across all 21 items. Values ≥0.78 for I-CVI and ≥0.90 for S-CVI were considered indicative of excellent content validity [8]. All items achieved an I-CVI ≥0.83, and the average S-CVI was 0.94, indicating excellent content validity.

4. Pilot Testing

4.1. Procedure
Following expert review, the revised FFQ was pilot tested with 20 participants (10 males, 10 females) recruited from the university community. Participants were representative of the target population (Saudi adults aged ≥18 years). Pilot testing was conducted using the same electronic Google Forms platform as the main study.

4.2. Pilot Objectives

- Assess face validity (participant understanding and ease of completion).

- Estimate average completion time for the FFQ section.
- Identify any ambiguous wording, missing food items, or technical issues.
- Test the functionality of the nine-point frequency scale in a digital format.

4.3. Pilot Results

- **Completion time:** The median time to complete the FFQ section was 6.5 minutes (range: 5–9 minutes).
- **Face validity:** All 20 participants reported that the instructions were clear, portion size descriptions were helpful, and the frequency options were easy to apply. No participants requested additional protein items.
- **Technical issues:** No technical or formatting issues were identified. All frequency options functioned correctly in Google Forms.
- **Minor revisions post-pilot:** Based on participant feedback, the term *"medium bowl serving"* for legumes was supplemented with *"½ cup"* for consistency. No items were added or removed.

- It is important to note that the pilot sample (n = 20) was sufficient to assess face validity and completion feasibility; it was not intended to support reliability estimation, internal consistency analysis, or factor analysis, which would require substantially larger samples. No psychometric claims beyond content and face validity are made because of this pilot.

5. Format of the Final FFQ

The final FFQ was presented as a table with the following columns:

- **No.** (1–21)
- **Food Item** (as listed in Section 2.1)
- **Standard Portion Size** (visual and metric descriptors)
- **Frequency Options** (drop-down menu with the nine-point scale)

The complete English version of the FFQ as administered to participants is provided in Supplementary File S1.

6. Limitations of the FFQ Validation

The present expert-reviewed, content-validated FFQ was assessed for content and face validity through expert review and pilot testing. However, it is important to acknowledge that: The FFQ was not subjected to relative validation against a reference dietary assessment method (e.g., 24-hour recall, weighed food records, or biomarkers). Consequently, quantitative intake estimates may be subject to recall bias and social desirability effects, and the reported carbon footprint values should be interpreted as indicative rather than precise measures of intake or emissions. Relative validity, construct validity, and test-retest reliability were not assessed in the present study. These represent important directions for future instrument development. This limitation is consistent with the targeted nature of the FFQ, which prioritized feasibility, respondent burden, and alignment with the study's primary environmental outcome (carbon footprint estimation) over comprehensive dietary assessment [1,2].

7. Full Food Frequency Questionnaire (FFQ) as Administered to Participants

How often do you consume the following foods? Please estimate the number of servings or your average intake of these foods as accurately as possible.

Please note that the frequency options refer to the number of servings consumed for each food item.

Total number of items in this section: 21

Frequency scale used for all FFQ items: 4 times/day; 3 times/day; 2 times/day; once/day; 5–6 times/week; 2–4 times/week; once/week; 1–3 times/month; never/rarely.

No.	Food Item	Standard Portion Size
	**Animal-Based Protein Sources**	
1	Lamb meat	100 g (standard cooked serving)
2	Beef / steak	100 g (standard cooked serving)
3	Ground meat products (burger, kebab, kofta)	100 g (standard cooked serving)
4	Organ meats (liver, kidney, heart, gizzard, brain)	100 g (standard cooked serving)
5	Chicken breast or thigh	100 g (standard cooked serving)
6	Whole egg	1 egg
7	Milk or laban	1 cup (240 mL)
8	Yogurt	170 g container
9	Cheese (feta, halloumi, cheddar, spread cheese)	30 g (small piece equivalent to two thumbs or three teaspoons)
10	Processed luncheon meat (mortadella, salami, turkey slices)	30 g (1–2 slices)
11	Sausages	1 piece (approximately 50 g)
12	Fresh fish / seafood	100 g (standard cooked serving)
13	Canned tuna	½ cup (approximately 80 g)
	**Plant-Based Protein Sources**	
14	Plant-based milk (almond, soy, coconut milk)	1 cup (240 mL)
15	Soybeans or soy-based protein alternatives (e.g., tofu)	½ cup (approximately 80–100 g cooked)
16	Chickpeas	½ cup (approximately 80–100 g cooked)
17	Beans	½ cup (approximately 80–100 g cooked)
18	Peas	½ cup (approximately 80–100 g cooked)
19	Lentils	½ cup (approximately 80–100 g cooked)
20	Fava beans	½ cup (approximately 80–100 g cooked)
21	Nuts (walnuts, almonds, peanuts)	30 g (approximately one handful)

Note. The FFQ was administered electronically using *Google Forms*. Frequency responses were collected through standardized drop-down menus. Portion size descriptors and household equivalents were displayed alongside each food item to facilitate participant recall and improve estimation accuracy. The FFQ was intentionally restricted to major protein-rich food sources to capture the primary contributors to dietary protein-related carbon footprint within the Saudi dietary context.

7. References

1. Willett, W.; Lenart, E. Reproducibility and validity of food-frequency questionnaires. In *Nutritional Epidemiology*, 3rd ed.; Oxford University Press: New York, NY, USA, 2013.
2. Kirkpatrick, S.I.; Baranowski, T.; Subar, A.F.; Tooze, J.A.; Frongillo, E.A. Best practices for conducting and interpreting studies to validate self-report dietary assessment methods. *J. Acad. Nutr. Diet.* **2019**, *119*, 1801–1816.
3. Gosadi, I.M.; Alatar, A.A.; Otayf, M.M.; AlJahani, D.M.; Ghabbani, H.M.; AlRajban, W.A.; Al-Nasser, K.A. Development of a Saudi Food Frequency Questionnaire and testing its reliability and validity. *Saudi Med. J.* **2017**, *38*, 636–643.
4. Ajabnoor, S.M.; Jambi, H.; Bahijri, S. Development and validation of a food frequency questionnaire in adult Saudi subjects in Jeddah city. *BMC Public Health* **2024**, *24*, 9.
5. Naja, F.; Jomaa, L.; Itani, L.; Zidek, J.; El Labban, S.; Sibai, A.M.; Hwalla, N. Environmental footprints of food consumption and dietary patterns among Lebanese adults: A cross-sectional study. *Nutr. J.* **2018**, *17*, 85.
6. British Nutrition Foundation. Nutrition Information: Protein. Available online: https://www.nutrition.org.uk (accessed on 29 January 2025).
7. Gaillac, R.; Marbach, S. The carbon footprint of meat and dairy proteins: A practical perspective to guide low carbon footprint dietary choices. *J. Clean. Prod.* **2021**, *321*, 128766.
8. Polit, D.F.; Beck, C.T. The content validity index: Are you sure you know what's being reported? Critique and recommendations. *Res. Nurs. Health* **2006**, *29*, 489–497.

Supplementary File S2: Calculation of the Carbon Footprint (CF) of Animal and Plant Protein Consumption

2.1. Overview of the Calculation Approach

The carbon footprint (CF) for each participant was calculated using a process-based, attributional Life Cycle Assessment (LCA) approach [1,2]. Emission factors represented cradle-to-gate or cradle-to-grave estimates depending on the original LCA source, as specified in Table 1 of the main manuscript. This method multiplies self-reported food consumption (in kilograms per day) by published peer-reviewed greenhouse gas (GHG) emission factors (expressed as kg of carbon dioxide equivalent per kilogram of food; $kgCO_2e/kg$). The sum across all 21 protein items provides an individual's daily dietary CF from protein sources. Saudi-specific food-system LCA coefficients are currently limited or unavailable for many protein categories; therefore, internationally peer-reviewed LCA datasets commonly used in dietary environmental assessment research were applied to maximize comparability with previous studies and to facilitate benchmarking against international evidence.

The general formula applied was:

- **$CF_{individual}$ ($kgCO_2e/day$) = Σ [$Consumption_i$ (kg/day) × EF_i ($kgCO_2e/kg$)]**
- Where:
- $CF_{individual}$ = Total daily carbon footprint for one participant.
- $Consumption_i$ = Daily intake of food item *i* (converted from FFQ frequency to kg/day).
- EF_i = The GHG emission factor for food item *i*.
- Σ = Summation across all 21 animal- and plant-based protein items.

2.2. Step-by-Step Calculation Procedure

Step 1: Convert FFQ frequency responses to daily intake (g/day)

For each of the 21 food items, participants selected one of nine frequency options. Each option was converted to a numeric value representing servings per week, then to grams per day.

Supplementary Table S1. Conversion of FFQ Frequency Categories into Standardized Weekly Consumption Values for Estimation of Daily Intake (g/day)

Frequency Option	Conversion to servings/week	Conversion factor
4 times/day	28	4 × 7 days
3 times/day	21	3 × 7 days
2 times/day	14	2 × 7 days
once/day	7	1 × 7 days
5–6 times/week	5.5	Midpoint (5+6)/2
2–4 times/week	3	Midpoint (2+4)/2
once/week	1	As stated

1–3 times/month	0.5	Midpoint assuming 2/month ÷ 4 weeks
never/rarely	0	0

Example calculation for lamb:

- A participant reports consuming lamb "2–4 times/week" (converted to 3 servings/week).
- Standard portion size for lamb = 100 g (from FFQ).
- Weekly intake = 3 servings × 100 g = 300 g/week.
- Daily intake = 300 g ÷ 7 days = 42.86 g/day.
- Convert to kg/day for CF calculation: 42.86 g ÷ 1000 = 0.04286 kg/day.

Step 2: Apply outlier management (data standardization)

As described in manuscript Section 2.3, an upper consumption cap of **35 servings/week** was applied to total animal and plant protein intake separately, following Hagmann et al. [3]. This cap was applied at the serving level before conversion to grams, not to the final CF value.

- Any participant reporting >35 servings/week of total animal protein items (summed across all 13 animal items) was capped at 35 servings/week.
- The same cap was applied symmetrically to plant protein items.
- This procedure minimizes the influence of extreme outliers while preserving the rank order of high consumers [4].

Step 3: Multiply daily consumption (kg) by emission factor (kgCO_2e/kg)

Each food item was assigned to a GHG emission factor derived from peer-reviewed LCA studies, as summarized in Table 1 of the main manuscript. Emission factors represent cradle-to-gate or cradle-to-grave emissions depending on the source, and include CO_2, CH_4, and N_2O expressed as CO_2 equivalents.

Example calculation for lamb (continuing from Step 1):

- Daily consumption = 0.04286 kg/day
- Emission factor for lamb = 69.00 kgCO_2e/kg (Opio et al., 2013) [5]
- CF contribution from lamb = 0.04286 kg/day × 69.00 kgCO_2e/kg = 2.96 kgCO_2e/day

Step 4: Sum across all 21 items for total daily CF

For each participant, the CF contributions from all 13 animal-based and 8 plant-based items were summed:

Total $CF_{day} = \Sigma(CF_{lamb} + CF_{beef} + CF_{minced\ meat} + \ldots + CF_{nuts})$

2.3. Emission Factors Used in This Study

The table below reproduces, including the corrected emission factors for luncheon meat, sausages, and yogurt (highlighted). All emission factors are expressed as kgCO_2e per kg of food product.

Supplementary Table S2. Standard portion sizes, protein per portion (g), and Life Cycle Assessment (LCA)-based greenhouse gas (GHG) emission factors of the 21 assessed protein-containing food items.

Protein source	Standard portion	Protein per portion (g)	Emission factor ($kgCO_2e/kg$)	Primary LCA source
Lamb	100 g	29.2	69.00	Opio et al., 2013 [5]
Beef	100 g	31.0	87.10	Heller et al., 2018 [6]
Minced (ground) meat	100 g	29.2	34.93	Naja et al., 2018 [7]
Organ meats	100 g	18.4	37.15	Opio et al., 2013 [5]
Luncheon meat	2 slices	20.6	5.98 (Revised - applied in main analysis)	Williams et al., 2006 [8] - processed meat LCA
Sausages	1 piece	11.7	7.61 (Revised - applied in main analysis)	Williams et al., 2006 [8] - processed meat LCA
Chicken breast	100 g	32.0	5.30	De Vries & De Boer, 2010 [9]
Egg	1 egg	14.1	4.00	Nijdam et al., 2012 [10]
Fresh fish	100 g	24.6	6.47	Samuel-Fitwi et al., 2013 [11]
Canned tuna	½ cup	12.45	6.47	Samuel-Fitwi et al., 2013 [11]
Milk	1 cup	4.3	1.50	Kim et al., 2013 [12]
Yogurt	1 container	9.4	2.20 (Revised - applied in main analysis)	Thoma et al., 2013 [13] - dairy-specific LCA
Cheese	30 g	9.0	13.50	Kim et al., 2013 [12]
Chickpeas	½ cup	7.6	1.50	Nijdam et al., 2012 [10]
Beans	½ cup	7.8	1.50	Nijdam et al., 2012 [10]
Peas	½ cup	5.4	1.50	Kim et al., 2013 [12]
Lentils	½ cup	9.0	1.50	Nijdam et al., 2012 [10]
Fava beans	½ cup	7.2	1.50	Nijdam et al., 2012 [10]
Tofu / soy alternatives	½ cup	8.1	3.72	Heller et al., 2018 [6]
Plant-based milk	1 cup	6.0	0.89	Heller et al., 2018 [6]
Nuts	1 handful	14.7	0.23	Esteve-Llorens et al., 2020 [14]

Note. LCA = Life Cycle Assessment; GHG = greenhouse gas; $kgCO_2e$ = kilograms of carbon dioxide equivalent. Emission factors were derived from peer-reviewed LCA-based studies selected for relevance to dietary and environmental assessment contexts. Values labeled "(Revised - applied in main analysis)" indicate emission factors corrected from the original analysis: luncheon meat (original: 69.0 $kgCO_2e/kg$; revised: 5.98 $kgCO_2e/kg$; Williams et al., 2006), sausages (original: 69.0 $kgCO_2e/kg$; revised: 7.61 $kgCO_2e/kg$; Williams et al., 2006), and yogurt (original: 14.0 $kgCO_2e/kg$; revised: 2.20 $kgCO_2e/kg$; Thoma et al., 2013). These corrected values are applied in all main manuscript tables, figures, and regression analyses; original values are retained here for reference only.

2.4. Aggregation Levels for Analysis

CF values were aggregated at three levels for statistical analysis:

1. **Item-specific CF ($kgCO_2e$/day):** For each of the 21 foods individually (presented in Table 4 of main manuscript).
2. **Animal vs. plant protein CF ($kgCO_2e$/day):** Sum of all 13 animal items vs. sum of all 8 plant items.
3. **Total dietary protein CF ($kgCO_2e$/day):** Sum of animal + plant CF.

2.5. Sensitivity Analysis for Minced (Ground) Meat

As noted in manuscript Section 2.5, the emission factor assigned to minced meat (34.93 $kgCO_2e$/kg) was substantially lower than that assigned to whole beef (87.1 $kgCO_2e$/kg). To evaluate the robustness of study findings to this assumption, a conservative sensitivity analysis was conducted in which the whole-beef emission factor was applied to minced/ground meat in place of the original coefficient. This analysis confirmed that qualitative findings were unchanged under both scenarios.

• Original method: Minced meat assigned 34.93 $kgCO_2e$/kg (from Naja et al. [7]).

• Sensitivity method: Minced meat assigned 87.1 $kgCO_2e$/kg (same as whole beef).

• Result for total sample: Mean animal-protein CF increased from 4.01 to 4.86 $kgCO_2e$/day, representing an approximate 21.2% increase.

• Consumption strata: Similar proportional increases were observed across low-, moderate-, and high-consumption groups.

• Qualitative conclusions: The principal findings of the study remained unchanged under both scenarios. Lamb and beef continued to represent the dominant contributors to total dietary CF, and the overall direction and magnitude of the animal-to-plant CF contrast (approximately 64-fold) were preserved.

Supplementary Table S3. Sensitivity Analysis for Minced Meat Emission Factor: Mean Daily Animal-Source Protein Food CF ($kgCO_2e$/day) Under Original and Conservative Scenarios.

Group	Original model (34.93 $kgCO_2e$/kg)	Conservative model (87.1 $kgCO_2e$/kg)	% Change
Total sample (N = 1,624)	4.01 ± 2.06	4.86 ± 2.49	**+21.2%**
Low consumers	1.82 ± 1.18	2.20 ± 1.43	**+20.9%**
Moderate consumers	4.06 ± 1.87	4.91 ± 2.26	**+20.9%**
High consumers	5.40 ± 1.64	6.54 ± 1.98	**+21.1%**

Note. Values are mean ± SD of daily animal-source protein food CF ($kgCO_2e$/day). The conservative model applies the whole-beef emission factor (87.1 $kgCO_2e$/kg) to minced/ground meat in place of the original Naja et al. coefficient (34.93 $kgCO_2e$/kg). The approximately 21% increase in CF was consistent across all consumption strata. Qualitative findings, relative rankings of protein sources, and the animal-to-plant CF ratio were unchanged under the conservative scenario.

2.6. Worked Example for One Participant

The table below provides a worked example for a hypothetical female participant in the *high-consumption category* (≥110 g animal protein/day). Items not listed were reported as "never/rarely" by this hypothetical participant and therefore contributed zero CF.

Supplementary Table S4. Worked Example of Carbon Footprint Calculation for a Hypothetical Participant

Food Item	FFQ Response	Servings/week	Daily intake (g)	Daily intake (kg)	EF ($kgCO_2e/kg$)	CF contribution ($kgCO_2e/day$)
Lamb	2–4/week	3	42.9	0.0429	69.00	2.96
Beef	once/day	7	100.0	0.1000	87.10	8.71
Chicken	2–4/week	3	42.9	0.0429	5.30	0.23
Egg	5–6/week	5.5	39.3*	0.0393	4.00	0.16
Milk	once/day	7	240.0[@]	0.2400	1.50	0.36
Chickpeas	once/week	1	17.9	0.0179	1.50	0.03
**Total**						**12.45**

*Assuming 1 egg = 50 g standard weight. [@]Assuming 1 cup = 240 mL milk.

2.7. Interpretation Note

The CF values reported in this study represent emissions from the 21 assessed protein items only and should be interpreted as a partial dietary CF estimate rather than a whole-diet CF. Other dietary components (grains, fruits, vegetables, beverages, fats, dates, composite mixed dishes) were not captured by the FFQ and may contribute additional dietary-related emissions not accounted for in the present analysis.

8. References

1. González-García, S.; Esteve-Llorens, X.; Moreira, M.T.; Feijoo, G. Carbon footprint and nutritional quality of different human dietary choices. *Sci. Total Environ.* **2018**, *644*, 77–94.
2. Van Dooren, C.; Aiking, H.; Vellinga, P. In search of indicators to assess the environmental impact of diets. *Int. J. Life Cycle Assess.* **2017**, *23*, 1297–1314.
3. Hagmann, D.; Siegrist, M.; Hartmann, C. Meat avoidance: Motives, alternative proteins and diet quality in a sample of Swiss consumers. *Public Health Nutr.* **2019**, *22*, 2448–2459.
4. Willett, W.; Lenart, E. Reproducibility and validity of food-frequency questionnaires. In *Nutritional Epidemiology*, 3rd ed.; Oxford University Press: New York, NY, USA, 2013.
5. Opio, C.; Gerber, P.; Mottet, A.; Falcucci, A.; Tempio, G.; MacLeod, M.; Vellinga, T.; Henderson, B.; Steinfeld, H. *Greenhouse Gas Emissions from Ruminant Supply Chains: A Global Life Cycle Assessment*; Food and Agriculture Organization of the United Nations: Rome, Italy, 2013.
6. Heller, M.C.; Willits-Smith, A.; Meyer, R.; Keoleian, G.A.; Rose, D. Greenhouse gas emissions and energy use associated with production of individual self-selected US diets. *Environ. Res. Lett.* **2018**, *13*, 044004.

7. Naja, F.; Jomaa, L.; Itani, L.; Zidek, J.; El Labban, S.; Sibai, A.M.; Hwalla, N. Environmental footprints of food consumption and dietary patterns among Lebanese adults: A cross-sectional study. *Nutr. J.* **2018**, *17*, 85.
8. Williams, A.G.; Audsley, E.; Sandars, D.L. *Determining the Environmental Burdens and Resource Use in the Production of Agricultural and Horticultural Commodities*; Main Report. Defra Research Project IS0205; Cranfield University and Defra: Bedford, UK, 2006.
9. de Vries, M.; de Boer, I.J.M. Comparing environmental impacts for livestock products: A review of life cycle assessments. *Livest. Sci.* **2010**, *128*, 1–11.
10. Nijdam, D.; Rood, T.; Westhoek, H. The price of protein: Review of land use and carbon footprints from life cycle assessments of animal food products and their substitutes. *Food Policy* **2012**, *37*, 760–770.
11. Samuel-Fitwi, B.; Nagel, F.; Meyer, S.; Schroeder, J.P.; Schulz, C. Comparative life cycle assessment (LCA) of raising rainbow trout (*Oncorhynchus mykiss*) in different production systems. *Aquac. Eng.* **2013**, *54*, 85–92.
12. Kim, D.; Thoma, G.; Nutter, D.; Milani, F.; Ulrich, R.; Norris, G. Life cycle assessment of cheese and whey production in the USA. *Int. J. Life Cycle Assess.* **2013**, *18*, 1019–1035.
13. Thoma, G.; Popp, J.; Nutter, D.; Shonnard, D.R.; Ulrich, R.; Matlock, M.; Kim, D.; Neiderman, Z.; Kemper, N.; East, C.; et al. Greenhouse gas emissions from milk production and consumption in the United States: A cradle-to-grave life cycle assessment circa 2008. *Int. Dairy J.* **2013**, *31*, S3–S14.
14. Esteve-Llorens, X.; Dias, A.C.; Moreira, M.T.; Feijoo, G.; González-García, S. Evaluating the Portuguese diet in the pursuit of a lower carbon and healthier consumption pattern. *Clim. Change* **2020**, *162*, 2397–2409.

2.8. Supplementary Regression Results: Sensitivity and Disaggregated Models

This section presents the complete results of two supplementary analyses conducted in response to peer review. Supplementary Table S4 shows the sensitivity analysis in which the 35 servings/week outlier cap was removed from animal-source protein food intake before re-estimating the sex-stratified models from Table 4 of the main manuscript. Supplementary Table S5 presents the disaggregated models in which animal-source protein food intake was separated into red meat (lamb + beef + minced meat + organ meats, g/day) and other animal proteins (all remaining animal items, g/day). Covariates in all models were identical to those in Table 4 of the main manuscript.

Supplementary Table S5. Sensitivity Analysis: Sex-Stratified Regression Coefficients With and Without the 35 Servings/Week Outlier Cap.

Model	B (95% CI)	β	p-value	R^2
Males (n = 765)				
Primary model (capped)	0.024 (0.019-0.029)	0.349	<0.001	0.273
Sensitivity model (uncapped)	0.023 (0.018-0.028)	0.341	<0.001	0.271
Females (n = 859)				
Primary model (capped)	0.019 (0.013-0.025)	0.254	<0.001	0.093
Sensitivity model (uncapped)	0.020 (0.014-0.026)	0.248	<0.001	0.091

Note. Dependent variable: daily carbon footprint (kgCO2e/day). B = unstandardized coefficient; CI = confidence interval. All models included the full covariate set from Table 4 of the main manuscript.

Supplementary Table S6. Disaggregated Regression Models: Red Meat and Other Animal Proteins as Separate Predictors of Daily Carbon Footprint.

Predictor	B (95% CI)	β	t	p-value
Males (n=765; R2=0.311; adj.R2=0.302; F=32.147; p<0.001)				
Red meat intake (g/day)	0.035 (0.029-0.041)	0.412	9.887	<0.001
Other animal protein (g/day)	0.009 (0.004-0.014)	0.118	2.981	0.002
Plant protein intake (g/day)	0.006 (0.001-0.011)	0.102	2.446	0.015
Age	0.038 (0.024-0.052)	0.179	5.234	<0.001
Diet type	0.218 (0.009-0.427)	0.066	2.041	0.042
Income	0.031 (-0.196, 0.258)	0.009	0.271	0.786
Weight	-0.001 (-0.017, 0.015)	-0.008	-0.122	0.903
BMI	0.014 (-0.044, 0.072)	0.034	0.471	0.638
Education	-0.038 (-0.242, 0.166)	-0.012	-0.362	0.718
Smoking	-0.091 (-0.376, 0.194)	-0.020	-0.629	0.530
Physical activity	-0.121 (-0.341, 0.099)	-0.036	-1.078	0.281
Females (n=859; R2=0.112; adj.R2=0.101; F=10.438; p<0.001)				
Red meat intake (g/day)	0.024 (0.017-0.031)	0.301	6.889	<0.001
Other animal protein (g/day)	0.004 (-0.002-0.010)	0.051	1.250	0.212
Plant protein intake (g/day)	0.003 (-0.001-0.007)	0.064	1.667	0.096
Age	0.004 (-0.009-0.017)	0.021	0.599	0.549
Diet type	0.005 (-0.163-0.173)	0.002	0.058	0.954
Income	0.118 (-0.055-0.291)	0.045	1.334	0.183
Weight	0.005 (-0.014-0.024)	0.022	0.516	0.606
BMI	-0.026 (-0.077-0.025)	-0.078	-0.999	0.318
Education	-0.015 (-0.186-0.156)	-0.006	-0.168	0.867
Smoking	0.059 (-0.287-0.405)	0.012	0.335	0.738
Physical activity	0.044 (-0.139-0.227)	0.016	0.464	0.643
Pooled model with interaction (n=1,624; R2=0.287; F=46.019; p<0.001)				
Red meat intake (g/day)	0.023 (0.017-0.029)	0.301	7.334	<0.001
Other animal protein (g/day)	0.006 (0.002-0.010)	0.071	2.775	0.006
Plant protein intake (g/day)	0.004 (0.001-0.007)	0.071	2.668	0.008
Sex (male=1)	0.421 (0.118-0.724)	0.102	2.724	0.007
Red meat x sex interaction	0.012 (0.003-0.021)	0.102	2.641	0.008
Age	0.021 (0.012-0.030)	0.110	4.517	<0.001
Diet type	0.112 (-0.034-0.258)	0.037	1.501	0.134
Income	0.092 (-0.043-0.227)	0.039	1.337	0.181
Weight	0.003 (-0.009-0.015)	0.014	0.471	0.638
BMI	-0.008 (-0.042-0.026)	-0.024	-0.464	0.643
Education	-0.024 (-0.149-0.101)	-0.011	-0.379	0.705
Smoking	-0.019 (-0.215-0.177)	-0.005	-0.192	0.848
Physical activity	-0.045 (-0.184-0.094)	-0.017	-0.628	0.530

Note. Red meat intake = lamb + beef + minced meat + organ meats (g/day). Other animal protein = chicken + eggs + milk + yogurt + cheese + luncheon + sausage + fish + tuna (g/day). All models included the full covariate set from Table 4 of the main manuscript.
